# ROLE OF THE METHYLTRANSFERASE METTL21C IN DRIVING MUSCLE ATROPHY WITH AGING AND HIV INFECTION

**DOI:** 10.1093/geroni/igad104.3579

**Published:** 2023-12-21

**Authors:** Jorge Gonzalez Castillo, Meghan McGee-Lawrence, Taylor Kress, Laszlo Kovacs, Eric Belin de Chantemele, Mark Hamrick

**Affiliations:** Universidad Central del Caribe, Bayamón, Puerto Rico, United States; Medical College of Georgia, Augusta, Georgia, United States; Medical College of Georgia, Augusta, Georgia, United States; Medical College of Georgia, Augusta, Georgia, United States; Medical College of Georgia, Augusta, Georgia, United States; Medical College of Georgia, Augusta, Georgia, United States

## Abstract

Osteoporosis and sarcopenia are both frequently observed in older people living with HIV. We developed an animal model to investigate the cellular and molecular mechanisms underlying accelerated muscle aging in the setting of HIV infection. Wild-type C57BL/6 mice received bone marrow from Tg26 HIV-1 transgenic mice at 8 weeks of age and tissues were collected 8 weeks later. Tg26 mice express 7 of 9 HIV proteins but lack gag and pol so are non-infectious. RNA was isolated from the tibialis anterior and microarray was performed using the Clariom S Mouse array that contains more than 22,100 well-annotated genes and 150,300 transcripts. Differential expressions were calculated using ANOVA and filtered with a p-value cutoff of 0.05. Results show that the methyltransferase Mettl21c, a methyltransferase previously shown to stimulate muscle protein synthesis and suppress muscle degradation, is one of the most highly downregulated (more than two-fold) genes in muscle from mice receiving Tg26 bone marrow. We then analyzed GEO data sets to identify changes in muscle Mettl21c expression in various disease settings. These data sets reveal that Mettl21c is consistently downregulated in skeletal muscle with inflammatory conditions such as cancer cachexia and sepsis. In addition, GWAS studies have previously shown Mettl21c deficiency in patients with sarcopenia and osteoporosis. Our data suggest that HIV proteins may produce epigenetic modifications in skeletal muscle leading to muscle loss, and that therapies restoring expression of methyltransferases such as Mettl21c might improve muscle health in patients living with HIV.

